# Primary giant splenic hydatid disease in a pregnant woman: case report

**DOI:** 10.17843/rpmesp.2022.394.12130

**Published:** 2022-12-22

**Authors:** Gino P. Segura-Gago, Rosita Estela-Reynel, Mónica Calisaya-Sánchez, Marlene Flores-Rodriguez

**Affiliations:** 1 Department of Gastroenterology, Hospital Nacional Hipólito Unanue, Lima, Peru. Department of Gastroenterology Hospital Nacional Hipólito Unanue Lima Perú; 2 Sociedad Científica de San Fernando. Universidad Nacional Mayor de San Marcos, Lima, Peru. Universidad Nacional Mayor de San Marcos Sociedad Científica de San Fernando Universidad Nacional Mayor de San Marcos Lima Peru; 3 Department of Clinical Pathology and Anatomical Pathology, Hospital Nacional Hipólito Unanue, Lima, Peru. Department of Clinical Pathology and Anatomical Pathology Hospital Nacional Hipólito Unanue Lima Peru

**Keywords:** Echinococcosis, Pregnancy, Spleen, Splenectomy, Fetal Growth Retardation

## Abstract

Hydatidosis is currently considered a public health problem in Peru. It is a parasitic infection transmitted by the ingestion of eggs of Echinococcus granulosus. The most involved organs are the liver and lungs, with spleen involvement being rare. We present the case of a young pregnant woman with abdominal pain and a sensation of mass in the left hypochondrium. The ultrasound study revealed a multiloculated cystic image in the left hemiabdomen, and a viable fetus. She underwent cesarean section, followed by exploratory laparotomy, which revealed a giant spleen tumor that, according to the anatomopathological study, corresponded to multicystic splenic hydatid disease. Likewise, intrauterine growth restriction was found as a fetal complication. The patient progressed favorably without recurrence of hydatid foci and the neonate had an adequate growth pattern.

## INTRODUCTION

Hydatidosis is a zoonotic disease currently considered an important public health problem in Peru, with a reported incidence of 100 cases per 100,000 inhabitants ^1^. Infection is acquired by ingestion of water or food contaminated with parasite eggs or by physical contact with host animals. The liver is the most frequently involved organ, followed by the lungs; on the other hand, the spleen is described as the most unusual location ^2^.

The growth pattern of splenic hydatidosis is slow and the diagnosis may go unnoticed for years, even more so if the disease is asymptomatic and the clinical manifestations are the result of a large lesion or even of some condition that may exacerbate the symptoms, such as pregnancy.

There is little information in the medical literature on the relationship between hydatid cyst, especially of purely splenic involvement, and the consequences on pregnancy and fetal development. Therefore, it is necessary to understand the causes that cause fetal disorders that are not attributed to gynecological or obstetric conditions.

We describe the case of a young pregnant woman diagnosed with hydatidosis that involved almost the entire spleen, and whose pregnancy outcome presented intrauterine growth restriction.

## CASE REPORT

A 19-year-old pregnant woman from the city of Jauja in Junín, with a history of previous pregnancy without complications and irregular attendance to her prenatal check-ups. She did not have previous history of threatened abortion during her current pregnancy. Her mother had been previously diagnosed with pulmonary hydatidosis that was treated. She presented sharp abdominal pain for four months, with sensation of a mass in the left hypochondrium, occasionally associated with dyspnea during inspiration and expiration.

She was admitted to the hospital due to exacerbation of symptoms with the following vital signs: blood pressure of 120/70 mmHg, heart rate of 60 per minute, respiratory rate of 16 per minute and temperature of 36.5 °C. Physical examination showed a pregnant abdomen, uterine height of 27 cm, fetal heart rate of 140 per minute, estimated weight by ultrasound of 2532 g (8th percentile), gestational age of 37 weeks and 2 days, with a diagnosis of intrauterine growth restriction. A palpable tumor was found in the left upper hemiabdomen of approximately 10 × 10 cm of hard consistency, mobile and not painful on palpation.

Laboratory results reported leukocytes: 17,400/uL, segmented neutrophils: 15,400/uL, hemoglobin: 14 g/dL, platelets: 12,2000/uL, C-reactive protein: 26.47 mg/dL, serum creatinine: 0.57 mg/dL, AST: 14.79 U/L, ALT: 5.19 U/L, total bilirubin: 0.27 mg/dL. Serologic tests for HIV, syphilis and hepatitis B were not reactive.

Transabdominal ultrasonographic evaluation showed an extensive spleen-dependent cystic image measuring 190 mm × 154 mm × 164 mm, with an approximate volume of 2500 cm^3^, thin and multiloculated walls, mass effect on the adjacent structures, very reduced splenic parenchyma, and without calcifications.

**Figure 1:**
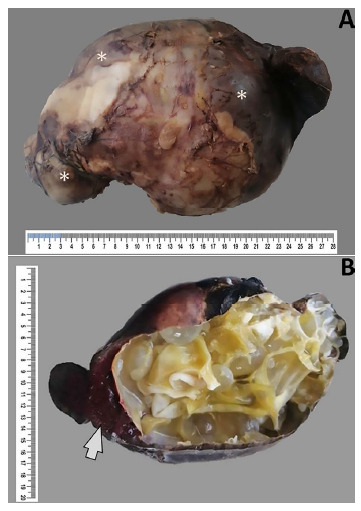
Pathologic anatomy: macroscopy of excluded spleen. A) Spleen with capsulated, vascularized, congestive, brownish-white external surface, with nodular areas (asterisks). B) Spleen with presence of multiple pearly white hydatid cysts involving 95% of the cut surface, with an area of splenic remnant of reddish-brown color and hemorrhagic aspect (arrow).

**Figure 2:**
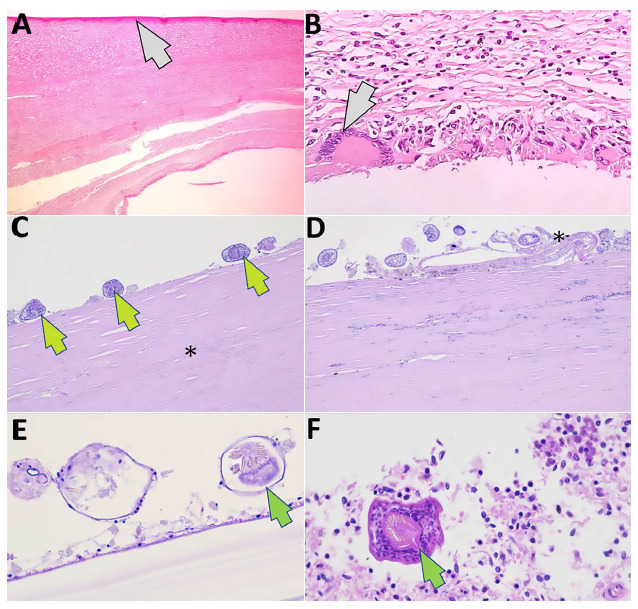
Pathologic anatomy: microscopy (histopathologic study) of excluded spleen. A) Splenic capsule (gray arrow) and fibroconjunctival tissue with chronic lymphomonocytic inflammatory infiltrate and abundant eosinophils (hematoxylin and eosin, 4X). B) Fibroconjunctival tissue as a host response with chronic inflammatory infiltrate and presence of macrophages, lymphocytes, eosinophils and multinucleated foreign body-like giant cells (gray arrow) (hematoxylin and eosin, 10X). C) Viable adventitial membrane (asterisk) and scolex (green arrows) (hematoxylin and eosin, 10X). D) Scarce germinative layer (asterisk) and scolex (hematoxylin and eosin, 40X). E) Germinative layer and scolex (green arrow) (hematoxylin and eosin, 100X). F) Hooked and sucker scolex (green arrow) (hematoxylin and eosin, 100X).

A 37-week newborn was born by cesarean section with a weight of 2225 g, head circumference of 32 cm, abdominal circumference of 27 cm, height of 43 cm and an Apgar score of 9. A surgical specimen with characteristics of a spleen-dependent abdominal tumor was obtained by exploratory laparotomy (Figure 1). The mass measured 28 cm × 17.5 cm × 12 cm, with translucent serous liquid content, and with multiple cystic structures inside. Histopathological evaluation of the spleen by hematoxylin and eosin staining showed numerous inflammatory cells and hydatid membranes with viable scolexes (Figure 2).

The patient evolved favorably. A CT scan of the thorax, abdomen and pelvis showed no involvement of the liver, lungs, or any other organ. She received prophylaxis with albendazole 400 mg orally every 12 h for 6 weeks, as well as pneumococcal and seasonal influenza vaccination. She was discharged without recurrence of hydatid foci during follow-up. The newborn had adequate weight and height gain, did not receive specific treatment, and was vaccinated according the vaccination schedule.

## DISCUSSION

We describe a rare manifestation of a parasitic disease in a young pregnant woman with no previous pathological history. The definitive management she received was cesarean section followed by splenectomy, which showed a fetal condition related to an active parasitic infection.

Hydatidosis is a zoonosis caused by the larvae of the *Echinococcus granulosus* species and it is endemic in Peru, mainly in the regions of Junín, Huancavelica, Cerro de Pasco, Cusco, Arequipa, and Puno, with an incidence of 100 cases per 100,000 inhabitants ^1^. It is considered a public health problem due to the combination of the lifestyle of the inhabitants, poor educational level, and precarious environmental sanitation. The coexistence of hydatidosis and pregnancy is a rare phenomenon, with an estimated one case per 20,000 to 30,000 pregnant women, which can increase morbidity and mortality rates ^3^.

Humans contract the disease by ingesting water and food contaminated with parasite eggs or physical contact with host animals (dogs). The parasite reaches the digestive tract and the liver through the portal venous circulation, and then it could travel to the pulmonary and systemic circulation, a situation in which any organ could be compromised ^4^.

The liver is usually the most affected organ (55-70%), followed by the lungs (18-35%), on the other hand, the spleen is only affected in 2% of cases ^2^. This tendency continues even during pregnancy. Splenic hydatidosis has a very slow growth rate that ranges from 0.3 cm to 1 cm per year in the general population ^5^, a figure that may vary during pregnancy, since, physiologically, there is a decrease in cellular immunity, which facilitates an accelerated growth of the parasite ^6^.

Signs vary, from the sensation of an abdominal mass in the left hypochondrium or epigastrium, constipation due to pressure on the colon or dyspnea due to its proximity to the diaphragm, left or right upper abdominal pain with dyspepsia or even no symptoms ^7^ ^,^ ^8^.

Serological diagnosis contributes only in certain cases, because of its’ high percentage of false negatives ^5^; on the other hand, abdominal ultrasonography is more useful due to its high sensitivity (90-95%) ^9^. Likewise, abdominal tomography can determine the location and measurements of the cyst, characteristics, anatomical areas of contact and show other possible lesions ^10^. However, it is contraindicated during pregnancy due to the risk of irradiation to the fetus.

Splenic hydatidosis can be associated with hepatic, pulmonary or multiorgan hydatidosis ^11^. In this case, a CT scan of the thorax, abdomen and pelvis was performed after surgery and did not show hepatic, pulmonary or other organ involvement, therefore it was considered a primary infection.

A study in Bulgaria found 40 cases of splenic hydatidosis; this included cases of extrapulmonary hydatidosis, of which only three presented two or more cysts, while the rest were solitary cysts ^12^. The internal exploration of the surgical specimen showed multiple cystic formations involving more than 90% of the structure, which is why it is considered a very infrequent presentation.

We considered epidermoid cyst, cystic neoplasia of the spleen, abscesses, hematomas, among others as differential diagnoses for the splenic lesion ^13^. Therefore, the anatomopathological study helped confirming the diagnosis due to the unusual nature of this case, considering the family history and the patient’s origin.

The complications of splenic hydatid disease are the infection of the cyst, formation of fistulas or rupture of neighboring organs and structures, therefore, patients may present pleural effusion, generalized abdominal pain, fever or symptoms of anaphylactic shock that could cause death ^14^. Cases of increased respiratory distress and chest pain due to the accelerated growth of the cyst, particularly in pregnant women, have been described in the literature; these cases may even lead to hydatid rupture, which requires an emergency cesarean section and resection of the cyst during the same surgical procedure ^15^. Regarding the fetal complications, a study in Turkey in 27 infected pregnant women reported cases of oligohydramnios, fetal distress, low birth weight, miscarriage, and premature placental abruption. The latter two corresponded to young pregnant women with splenic involvement hydatidosis, with cysts larger than 10 cm in diameter. In addition, most of the fetal complications were related to the presence of active cysts type I, II and III of Gharbi’s classification, obtained by transabdominal ultrasound ^16^. In our case, the multicystic appearance of the splenic lesion (which would correspond to Gharbi type III) and the finding of viable scolexes could be related to fetal intrauterine growth restriction, therefore, further studies are needed.

Vertical transmission of hydatidosis did not occur in our case. In this regard, a study in Chile in nine infected pregnant women who received cyst resection surgery, found that none of the newborn were serologically positive for hydatidosis ^17^.

Benzimidazoles, such as albendazole, are drugs commonly used in the management of the disease. It is necessary to identify their teratogenic and embryotoxic potential, which has been previously described in rats and rabbits, and is the reason why they are not recommended during the first trimester of gestation. Nevertheless, it could be used from the second trimester onwards, especially for the treatment of rapidly growing hydatid cysts ^16^. In general, it is recommended to associate albendazole one month prior to surgery and one month after surgery, with the intention of reducing the volume of the cyst, facilitating surgery, and reducing the risk of secondary cyst formation by sterilizing the disseminated scolexes ^18^.

Surgery is considered to be the main form of treatment, and it is recommended to wait until 20 to 24 weeks of gestation to allow fetal maturation and reevaluate the cysts ^16^. Surgical options include total splenectomy or other more conservative alternatives such as unroofing with omentoplasty, pericystectomy, internal cystojejunal anastomosis or partial splenectomy ^7^. Total splenectomy allows complete eradication of the parasite and avoids relapses, although this technique is preferred to be reserved for multiple cysts or those that destroy more than 75% of the splenic parenchyma ^14^. This can considerably reduce the incidence of post-splenectomy infections, as well as thromboembolic complications ^19^. On the other hand, surgery has been considered to can interfere with the normal labor process ^6^, which is why cesarean section is being recommended.

One of the limitations of this case report is that a serological study, specific for hydatidosis, was not performed. Neither was it possible to access the printed image of the abdominal ultrasound that would have helped to define more specific characteristics of the splenic lesion. Nevertheless, the anatomopathological study was able to demonstrate the existence of the infection, as well as the viability of the cysts.

We conclude that splenic hydatidosis during pregnancy is an unusual presentation documented in Peru, where this parasitic infection is considered an important public health problem. The growth of the lesion can be altered and the clinical presentation is nonspecific or asymptomatic. Diagnosis is clinical, serological, by imaging, and histopathological, depending on the case. Fetal complications have been described and are related to the activity of the cyst. Management is mainly surgical and the association with antiparasitic drugs such as albendazole may be recommended considering the gestational age. We consider that the described case will help to raise awareness of the maternal-fetal consequences caused by a zoonosis of singular manifestation. Likewise, it is necessary to sensitize and educate the population in order to develop hygiene awareness from an early age as a precautionary measure against the disease.
