# Supplementary material for: Primary giant splenic hydatid disease in a pregnant woman: case report
Source: Rev Peru Med Exp Salud Publica. 2022 Dec 22;39(4):495–9. doi: 10.17843/rpmesp.2022.394.12130 (PMC11397591; doi:10.17843/rpmesp.2022.394.12130)
Supplement: Translation

# Translation: Hidatidosis esplénica gigante primaria en una gestante: reporte de caso

**Authors:** Gino P. Segura-Gago, Rosita Estela-Reynel, Mónica Calisaya-Sánchez, Marlene Flores-Rodriguez

**Affiliations:** 1 Departamento de Gastroenterología, Hospital Nacional Hipólito Unanue, Lima, Perú.; 2 Sociedad Científica de San Fernando. Universidad Nacional Mayor de San Marcos, Lima, Perú.; 3 Departamento de Patología Clínica y Anatomía Patológica, Hospital Nacional Hipólito Unanue, Lima, Perú.

**Keywords:** Equinococosis, Esplenectomía, Embarazo, Esplenomegalia, Retardo del Crecimiento Fetal

## Abstract

La hidatidosis es considerada en la actualidad como un problema de salud pública en el Perú. Es una infección parasitaria trasmitida por la ingesta de huevos de *Echinococcus granulosus *y los órganos más comprometidos son el hígado y los pulmones, siendo rara la afectación del bazo. Presentamos el caso de una gestante joven con dolor abdominal y sensación de masa en hipocondrio izquierdo. El estudio ecográfico reveló imagen quística multitabicada en hemiabdomen izquierdo, con feto viable. Se le realizó cesárea, seguido de laparotomía exploratoria y se halló una tumoración gigante de bazo que, según el estudio anatomopatológico corresponde a una hidatidosis esplénica multiquística. Asimismo, como complicación fetal ocurrió restricción del crecimiento intrauterino. La paciente evolucionó favorablemente sin recurrencia de focos hidatídicos, mientras que el neonato mostró un patrón de crecimiento adecuado.

## INTRODUCCIÓN

La hidatidosis es una zoonosis considerada en la actualidad como un importante problema de salud pública en el Perú, reportándose una incidencia de 100 casos por cada 100 00 habitantes ^1^. La infección se adquiere por la ingesta de agua o alimentos contaminados con huevos del parásito o por contacto físico con animales hospederos. Los órganos más frecuentemente comprometidos son el hígado seguido del pulmón, mientras que la localización esplénica de la infección está descrita como inhabitual ^2^. 

El patrón de crecimiento de la hidatidosis esplénica es lento y el diagnóstico puede pasar inadvertido por años, más aún si la enfermedad cursa de forma asintomática y las manifestaciones clínicas son resultado de una lesión de gran volumen o incluso de alguna condición que pueda exacerbar los síntomas, como el embarazo.

Existe poca información en la literatura médica sobre la relación entre el quiste hidatídico, especialmente del compromiso exclusivamente esplénico, y las consecuencias en el embarazo y el desarrollo fetal. Por ello, es necesario conocer las causas que ocasionan problemas fetales y que no sean atribuidas a condiciones ginecológicas u obstétricas.

Describimos el caso de una gestante joven con diagnóstico de hidatidosis que comprometió casi la totalidad del bazo, y cuyo producto final de la gestación presentó restricción del crecimiento intrauterino.

## REPORTE DEL CASO

Gestante de 19 años procedente de la ciudad de Jauja en Junín, con antecedente de gestación anterior sin complicaciones y asistencia irregular a sus controles prenatales. Sin historial previo de amenaza de aborto en su gestación actual. Con antecedente familiar de madre con diagnóstico de hidatidosis pulmonar tratada. Presentó dolor abdominal tipo hincada desde hace cuatro meses, con sensación de masa en hipocondrio izquierdo que, ocasionalmente se asoció con disnea durante la inspiración y espiración. 

Ingresó al hospital por exacerbación de síntomas con funciones vitales: presión arterial de 120/70 mmHg, frecuencia cardiaca de 60 por min, frecuencia respiratoria de 16 por min, temperatura de 36,5 °C. Al examen físico se evidenció abdomen grávido, altura uterina de 27 cm, latidos cardiacos fetales de 140 por min, peso estimado por ecografía de 2532 g (percentil 8) con edad gestacional de 37 semanas y 2 días, con diagnóstico de restricción de crecimiento intrauterino. Presencia de tumoración palpable en hemiabdomen superior izquierdo de aproximadamente 10 cm × 10 cm de consistencia dura, móvil y no doloroso a la palpación. 

El estudio de laboratorio informó leucocitos: 17 400/uL, segmentados: 15 400/uL, hemoglobina: 14 g/dL, plaquetas: 12 2000/uL, proteína C reactiva: 26,47 mg/dL, creatinina sérica: 0,57 mg/dL, TGO: 14,79 U/L, TGP: 5,19 U/L, bilirrubina total: 0,27 mg/dL. Se realizó estudio serológico para VIH, sífilis y hepatitis B siendo no reactivos.

La evaluación ultrasonográfica transabdominal evidenció una imagen extensa quística dependiente del bazo que midió 190 mm × 154 mm × 164 mm, con volumen aproximado de 2500 cm^3^, de paredes delgadas multitabicadas con efecto de masa sobre estructuras adyacentes, parénquima esplénico muy reducido, sin calcificaciones.

La cesárea permitió obtener un producto de 37 semanas según Capurro, Apgar 9/9, con peso de 2225 g, perímetro cefálico de 32 cm, abdominal de 27 cm, talla de 43 cm. Luego se procedió a laparotomía exploratoria donde se obtuvo una pieza quirúrgica con características de tumoración abdominal dependiente del bazo (Figura 1). Las medidas fueron 28 cm × 17,5 cm × 12 cm, con contenido líquido seroso traslúcido al corte trasversal, y con formaciones quísticas múltiples en su interior. En la histopatología del bazo mediante tinción de hematoxilina y eosina se encontraron numerosas células inflamatorias y membranas hidatídicas con escólex viables (Figura 2).

**Figura 1:** Anatomía patológica: macroscopía de bazo excluido. **A) **Bazo de superficie externa capsulada, vascularizada, congestiva, color blanquecino parduzco, con áreas nodulares (asteriscos). **B)** Bazo con presencia de múltiples quistes hidatídicos blanco nacaradas que comprometen el 95% de la superficie del corte, con área de remanente esplénico de color pardo rojizo y aspecto hemorrágico (flecha).

**Figura 2:** Anatomía patológica: microscopía (estudio histopatológico) de bazo excluido.** A) **Se observa la cápsula esplénica (flecha gris) y tejido fibroconjuntivo con infiltrado inflamatorio crónico linfomonocitario y abundantes eosinófilos (hematoxilina y eosina, 4X).** B)** Tejido fibroconjuntivo como respuesta del huésped con infiltrado inflamatorio crónico y presencia de macrófagos, linfocitos, eosinófilos y células gigantes multinucleadas tipo cuerpo extraño (flecha gris) (hematoxilina y eosina, 10X).** C) **Membrana adventicia (asterisco) y escólex viables (flechas verdes) (hematoxilina y eosina, 10X). **D)** Escasa capa germinativa (asterisco) y escólex (hematoxilina y eosina, 40X). **E)** Capa germinativa y escólex (flecha verde) (hematoxilina y eosina, 100X). F) Escólex con ganchos y ventosa (flecha verde) (hematoxilina y eosina, 100X).

La paciente evolucionó satisfactoriamente. Se le realizó tomografía computarizada de tórax, abdomen y pelvis, donde no se encontró compromiso hepático, pulmonar, ni en el resto de los órganos. Recibió profilaxis con albendazol 400 mg vía oral cada 12 h por 6 semanas, vacunación antineumocócica y contra influenza estacional. Fue dada de alta sin observarse recurrencia de focos hidatídicos durante el seguimiento. El neonato tuvo ganancia adecuada de peso y talla, no recibió tratamiento específico y recibió su vacunación de acuerdo con sus meses de vida.

## DISCUSIÓN

Se describe una manifestación infrecuente de una enfermedad parasitaria en una gestante joven sin antecedentes patológicos previos. El manejo definitivo que recibió fue la cesárea seguida de la esplenectomía en los cuales se evidenció una afección fetal relacionada con una infección parasitaria activa.

La hidatidosis constituye una zoonosis ocasionada por la larva de la especie* Echinococcus granulosus,* endémica en el Perú, principalmente de las regiones de Junín, Huancavelica, Cerro de Pasco, Cusco, Arequipa y Puno, con una incidencia de 100 casos por cada 100 000 habitantes ^1^. Se considera un problema de salud pública al confluir el estilo de vida de los habitantes, deficiente nivel educativo y un precario saneamiento ambiental. La coexistencia entre la hidatidosis y la gestación es un fenómeno raro, estimándose un caso de cada 20 000 a 30 000 mujeres embarazadas, pudiendo elevar las cifras de morbimortalidad ^3^.

El ser humano contrae la enfermedad por la ingesta de agua y alimentos contaminados con huevos del parásito o contacto físico con animales hospederos (perros). El parásito al llegar al tubo digestivo y alcanzar el hígado mediante la circulación venosa portal podría transitar hacia la circulación pulmonar y sistémica, situación en que cualquier órgano puede verse comprometido^ (^ ^4^. 

El hígado constituye el órgano más infectado (55-70%), seguido del pulmón (18-35%), mientras que la localización en el bazo es infrecuente encontrándose en solo 2% de los casos ^2^. Esta tendencia se mantiene, inclusive, durante el embarazo. La hidatidosis esplénica presenta una tasa de crecimiento muy lenta que oscila entre 0,3 cm a 1 cm por año en la población general ^5^, cifra que durante la gestación puede variar, ya que, fisiológicamente, existe una disminución de la inmunidad celular, que facilita un crecimiento acelerado del parásito ^6^. 

Los signos varían, desde la sensación de una masa abdominal en hipocondrio izquierdo o epigastrio, estreñimiento por presión en el colon o disnea por su cercanía al diafragma, dolor abdominal superior izquierdo o derecho con dispepsia o inclusive cursar sin síntomas^ (^ ^7^ ^,^ ^8^.

La serología contribuye solo en determinados casos, por presentar un porcentaje elevado de falsos negativos^ (^ ^5^, siendo de mayor utilidad la ultrasonografía abdominal por su elevada sensibilidad (90-95%) ^9^. Asimismo, la tomografía abdominal puede determinar la localización y medidas del quiste, características, contactos anatómicos y mostrar otras posibles lesiones ^10^. Sin embargo, está contraindicado realizarla durante la gestación por el riesgo de irradiación al feto.

La hidatidosis esplénica puede asociarse con hidatidosis hepática, pulmonar o multiorgánica^ (^ ^11^. En nuestro caso, posterior a la cirugía, se realizó una tomografía de tórax, abdomen y pelvis donde no se evidenció compromiso hepático, pulmonar ni del resto de órganos, considerándose por ello como una infección primaria.

En un estudio en Bulgaria, en donde se recopilaron casos de hidatidosis extrapulmonar se encontraron 40 de hidatidosis esplénica, de las cuales solo tres presentaron de dos o más quistes, mientras que el resto fueron quistes solitarios ^12^. La exploración interna de la pieza quirúrgica mostró múltiples formaciones quísticas que comprometen más del 90% de la estructura, por lo que se considera como una presentación muy infrecuente.

Como diagnósticos diferenciales de la lesión esplénica se considera el quiste epidermoide, la neoplasia quística de bazo, abscesos, hematomas, entre otros^ (^ ^13^. Por ello, ante lo inusual del caso presentado, el estudio anatomopatológico permitió corroborar el diagnóstico, teniendo en cuenta los antecedentes familiares y la procedencia de la paciente.

Las complicaciones de la hidatidosis esplénica son la infección del quiste, formación de fístulas o ruptura de órganos y estructuras vecinas, por lo que los pacientes pueden presentar efusión pleural, dolor abdominal generalizado, fiebre o síntomas de shock anafiláctico que podría causar la muerte ^14^. En las gestantes en particular se ha descrito en la literatura casos de incremento de dificultad respiratoria y dolor torácico debido al crecimiento acelerado del quiste, que llega incluso a la ruptura hidatídica donde se requirió una cesárea de emergencia y resección del quiste en la misma sesión quirúrgica ^15^. Dentro de las complicaciones fetales, un estudio en Turquía en 27 gestantes infectadas reportó casos de oligohidramnios, sufrimiento fetal, bajo peso al nacer, aborto y desprendimiento prematuro de placenta. Estos dos últimos correspondieron a gestantes jóvenes con hidatidosis de compromiso esplénico, con quistes de diámetro mayor a 10 cm. Además, la mayoría de las complicaciones fetales estuvo relacionada con la presencia de quistes activos tipo I, II y III de la clasificación de Gharbi, obtenida mediante ecografía transabdominal ^16^. En el nuestro caso, la apariencia multiquística de la lesión esplénica (que correspondería al tipo Gharbi III) y el hallazgo de escólex viables podrían estar relacionados con la restricción de crecimiento intrauterino fetal, por lo que se necesitarían mayores estudios al respecto.

La transmisión vertical de la hidatidosis no se halló en nuestro caso. Referente a ello, en un estudio en Chile en nueve mujeres gestantes infectadas que recibieron cirugía de resección de quistes se verificó que ninguno de los bebés resultó serológicamente positivo para hidatidosis ^17^.

Los fármacos comúnmente utilizados en el manejo de la enfermedad son los benzimidazoles, como el albendazol. Es necesario precisar su potencial teratogénico y embriotóxico demostrado en ratas y conejos, por lo cual se desaconseja su administración durante el primer trimestre de gestación. A pesar de ello, podría usarse a partir del segundo trimestre, especialmente para el tratamiento de quistes hidatídicos de crecimiento rápido ^16^. En general, se recomienda asociar albendazol un mes previo a la cirugía y un mes posterior a ella, con la intención de reducir el volumen del quiste, facilitando la cirugía y disminuyendo el riesgo de formación de quistes secundarios mediante la esterilización de los escólex diseminados ^18^. 

El tratamiento es fundamentalmente quirúrgico y se recomienda esperar hasta las 20 a 24 semanas de gestación para permitir la maduración fetal y reevaluar los quistes ^16^. Como opciones quirúrgicas se menciona la esplenectomía total u otras alternativas más conservadoras como el destechamiento con omentoplastía, periquistectomía, anastomosis cistoyeyunal interna o la esplenectomía parcial ^7^. La esplenectomía total permite la erradicación completa del parásito y evita la aparición de recidivas, aunque se prefiere reservar esta técnica en quistes múltiples o aquellos que destruyen más del 75% del parénquima esplénico ^14^. De esta forma se reduce considerablemente la incidencia de infecciones posesplenectomía, así como de complicaciones tromboembólicas ^19^. Por otro lado, se ha considerado que la cirugía puede interferir con el proceso de trabajo de parto normal ^6^, por lo cual la cesárea está siendo recomendada.

Como limitaciones del presente reporte se debe reconocer que no se realizó un estudio serológico dirigido a la hidatidosis. Tampoco fue posible acceder a la imagen impresa de la ecografía abdominal que hubiera ayudado a definir características más específicas de la lesión esplénica. No obstante, el estudio anatomopatológico pudo demostrar la existencia de la infección, así como la viabilidad de los quistes.

Se concluye, que la hidatidosis de localización esplénica durante la gestación constituye una presentación inusualmente documentada en el Perú en donde esta infección parasitaria es considerada un importante problema de salud pública. El crecimiento de la lesión puede alterarse y la presentación clínica cursa de forma inespecífica o asintomática. El diagnóstico es clínico, serológico, imagenológico e histopatológico, según sea el caso. Las complicaciones fetales se han descrito y guardan relación con la actividad del quiste. El manejo es fundamentalmente quirúrgico y la asociación con antiparasitarios como el albendazol puede estar recomendada considerando el trimestre de gestación. Consideramos que el caso descrito ayudará a concientizar sobre las consecuencias materno-fetales ocasionadas por una zoonosis de manifestación singular. Asimismo, es necesario sensibilizar y educar a la población a desarrollar una conciencia de higiene desde edades tempranas como medida de precaución contra la enfermedad.
